# Incidence of Complicated Appendicitis during the COVID-19 Pandemic versus the Pre-Pandemic Period: A Systematic Review and Meta-Analysis of 2782 Pediatric Appendectomies

**DOI:** 10.3390/diagnostics12010127

**Published:** 2022-01-06

**Authors:** Zenon Pogorelić, Sachit Anand, Tomislav Žuvela, Apoorv Singh, Zvonimir Križanac, Nellai Krishnan

**Affiliations:** 1 Department of Pediatric Surgery, University Hospital of Split, 21 000 Split, Croatia; 2 Department of Surgery, School of Medicine, University of Split, 21 000 Split, Croatia; tomislav.zuvela29@gmail.com; 3 Department of Pediatric Surgery, Kokilaben Dhirubhai Ambani Hospital, Mumbai 400053, India; kanusachit@gmail.com; 4 Department of Pediatric Surgery, All India Institute of Medical Sciences, New Delhi 110029, India; dr.singhapoorv@gmail.com (A.S.); nellai93@gmail.com (N.K.); 5 Department of Surgery, University Hospital of Split, 21 000 Split, Croatia; zvonimir.krizanac@gmail.com

**Keywords:** acute appendicitis, appendectomy, Coronavirus Disease 2019, COVID-19, non-operative management, children

## Abstract

Background: The Coronavirus Disease 2019 (COVID-19) pandemic has impacted volume, management strategies and patient outcomes of acute appendicitis. The aim of this systematic review and meta-analysis was to evaluate whether the COVID-19 pandemic resulted in higher incidence of complicated appendicitis in children presenting with acute appendicitis compared to the pre-COVID-19 period. The secondary aim was to investigate the proportion of the patients treated by non-operative management (NOM). Methods: A systematic search of four scientific databases was performed. The search terms used were (coronavirus OR SARS-CoV-2 OR COVID-19 OR novel coronavirus) AND (appendicitis). The inclusion criteria were all patients aged <18 years and diagnosed with acute appendicitis during the COVID-19 and pre-COVID-19 periods. The proportion of children presenting with complicated appendicitis and the proportion of children managed by NOM was compared between the two groups. The Downs and Black scale was used for methodological quality assessment. Results: The present meta-analysis included thirteen studies (twelve retrospective studies and one cross-sectional study). A total of 2782 patients (1239 during the COVID-19 period) were included. A significantly higher incidence of complicated appendicitis (RR = 1.63, 95% CI 1.33–2.01, *p* < 0.00001) and a significantly higher proportion of children managed via the NOM (RR = 1.95, 95% CI 1.45–2.61, *p* < 0.00001) was observed in patients during the COVID-19 pandemic when compared to the pre-COVID-19 period. Conclusion: There is a significantly higher incidence of complicated appendicitis in children during the COVID-19 pandemic than in the pre-COVID-19 period. Additionally, a significantly higher proportion of children was managed via the NOM during the pandemic in comparison to the pre-pandemic period.

## 1. Introduction

Acute appendicitis is the most common condition in the pediatric population that leads to emergency abdominal surgery [1,2]. Although advanced diagnostic imaging is widely available, the initial diagnosis of appendicitis in children can be challenging, with rates of misdiagnosis reaching 100% in children aged two years or younger [3,4,5,6]. This has been attributed to nonspecific presentation and overlap of symptoms with other common childhood conditions such as mesenteric lymphadenitis, gastroenteritis, or Meckel’s diverticulitis. Clinical scores, such as Alvarado, appendicitis inflammatory response score, and pediatric appendicitis score have been developed to aid the diagnosis of acute appendicitis in children [3,4]. The diagnostic delay often leads to a higher incidence of complications, such as perforation. Perforation rates show an inverse relation to age, ranging from 47.3% in children five years of age, to 100% in children under two years of age [5,6]. Elevated inflammatory markers from blood or even hyponatremia and hyperbilirubinemia have been shown to assist in distinguishing between simple and perforated appendicitis [7,8,9]. Despite advances in medicine, especially in imaging diagnostics, acute appendicitis still in a certain percentage of patients remains unrecognized and mistreated in the initial stage of the disease [1,8,9].

In addition to all diagnostic challenges, the Coronavirus Disease 2019 (COVID-19) pandemic has become a new obstacle to overcome. The pandemic has disrupted the normal practice of economy, governance, and scientific and medical expertise [10]. Confinement measures, introduced in order to minimize the number of infected people, have had an impact on patients, medical procedures, and healthcare workers [11]. Many governing bodies have recommended the cancellation of elective surgical procedures during the pandemic, resulting in a major burden on healthcare systems [12]. A decline in admission rates for numerous medical and surgical conditions has been observed, possibly due to a generalized public fear of presenting to a hospital during the pandemic [13,14,15,16,17]. Despite the confinement measures, acute appendicitis does not quarantine [18]. The pandemic has impacted volume, diagnostic and management strategies, and patient outcomes of acute appendicitis [19,20]. A nationwide study in the United States found a significant decrease in acute appendicitis presentation, while two studies from Germany observed a decrease in the number of appendectomies during the lockdown [19,21,22]. Additionally, it was suggested that non-operative management (NOM) could be a safe alternative to surgery during the pandemic [23,24,25]. A study from Budapest suggests that a higher number of perforated appendices is in line with international trends, and shares no correlation with the COVID-19 pandemic [24]. In contrast, various studies have also demonstrated no significant differences in the rates of complicated appendicitis among children presenting during the pandemic versus the pre-pandemic period [19,20]. Due to these conflicting findings, there is no consensus statement regarding the incidence of complicated appendicitis among the children presenting during the pandemic.

The aim of this study was to systematically summarize and compare all relevant data on pediatric complicated appendicitis during the pandemic and pre-pandemic periods. A secondary aim was to investigate the proportion of the patients treated by non-operative management (NOM). We hypothesize that the incidence of complicated appendicitis is higher in children presenting during the COVID-19 pandemic.

## 2. Materials and Methods

### 2.1. Systematic Search

The present review was not registered in any prospective register or database. The systematic search was conducted in accordance with the Preferred Reporting Items for Systematic Reviews and Meta-Analyses (PRISMA) guidelines [26]. A pilot literature search in the PubMed database was independently performed by two authors (SA and NK) on 14 October 2021, to confirm the absence of any systematic reviews on this subject. On the same day, both the authors systematically explored the PubMed, EMBASE, Web of Science, and Scopus databases (Appendix A, Table A1) using the following keywords: (Coronavirus OR SARS-CoV-2 OR COVID-19 OR novel Coronavirus) AND (appendicitis). Search filters were used to identify the studies in the pediatric population (Appendix A). Following identification of the total records and duplication removal, the remaining articles were screened as per the inclusion/exclusion criteria to select the relevant ones.

### 2.2. Eligibility Criteria

The inclusion criteria in the present systematic review were: Participants—All patients aged <18 years and diagnosed with acute appendicitis (clinico-radiologic criteria) during the COVID-19 pandemic; Intervention—appendectomy or conservative management; Comparison—patients with acute appendicitis presenting during the pre-pandemic period (similar months of the previous year); Outcomes—the proportion of children presenting with complicated appendicitis (perforation of appendix, or gangrenous appendix, or abscess/phlegmon) was the primary outcome studied in this review. The proportion of children managed by NOM was the secondary outcome.

All comparative studies reporting the primary outcome were eligible for inclusion in the meta-analysis. The type of operative approach, whether laparoscopic or open appendectomy, was not considered a specific eligibility criterion in this review. During the process of selection of the studies for meta-analysis, articles in non-English language were translated using Google translate feature of the Google search engine. Studies with unavailable full-texts were excluded. In addition, case reports, case series, expert opinions, editorials, review articles, and commentaries were also excluded.

### 2.3. Data Synthesis

Data extraction was independently performed by two authors (T.Ž. and Z.K.) utilizing Microsoft Excel spreadsheets (Version 15.24). Data including the total sample size, the number of patients in each treatment group, the average duration of symptoms, the proportion of children with complicated appendicitis, the proportion of children managed by the NOM, and the average length of hospital stay (LOS) were recorded. In addition, the bibliographic information of each included study, i.e., the name of the first author, year of publication, and the type of the study design were also recorded. Any disagreements among the authors were resolved by discussion with the senior author (Z.P.).

### 2.4. Methodological Quality Assessment

Two authors (S.A. and A.S.) independently performed the methodological quality assessment utilizing the Downs and Black scale [27]. This twenty-seven-item scale has minimum and maximum scores of 0 and 32, respectively. On the basis of these scores, the risk of bias in each study was declared as high (score 0–15), moderate (score = 16–23), or low (score > 23). Subsequently, the inter-observer agreement on these scores was adjudged using the Kappa statistics [28]. The level of agreement was defined as almost perfect (0.81–1.00), substantial (0.61–0.80), moderate (0.41–0.60), fair (0.21–0.40), or slight (0.00–0.20).

### 2.5. Data Analysis

The baseline data were expressed as numbers, proportions, averages (mean or median), and ranges. Meta-analysis was performed using the RevMan 5.4 (Cochrane Collaboration, London, UK) software. As both the outcomes (primary and secondary) were dichotomous, the risk ratios (with 95% CI) were calculated for them. Subsequently, the Mantel–Haenszel method was used to evaluate the pooled risk ratio [29]. Heterogeneity among the included studies was estimated using the I^2^ statistics. A random-effects model was selected if the heterogeneity was substantial (I^2^ > 50%). A *p*-value of <0.05 was considered statistically significant. For the purpose of analysis, groups A and B included children presenting during the COVID-19 pandemic and pre-pandemic periods, respectively.

## 3. Results

### 3.1. Study Characteristics

**Figure 1:**
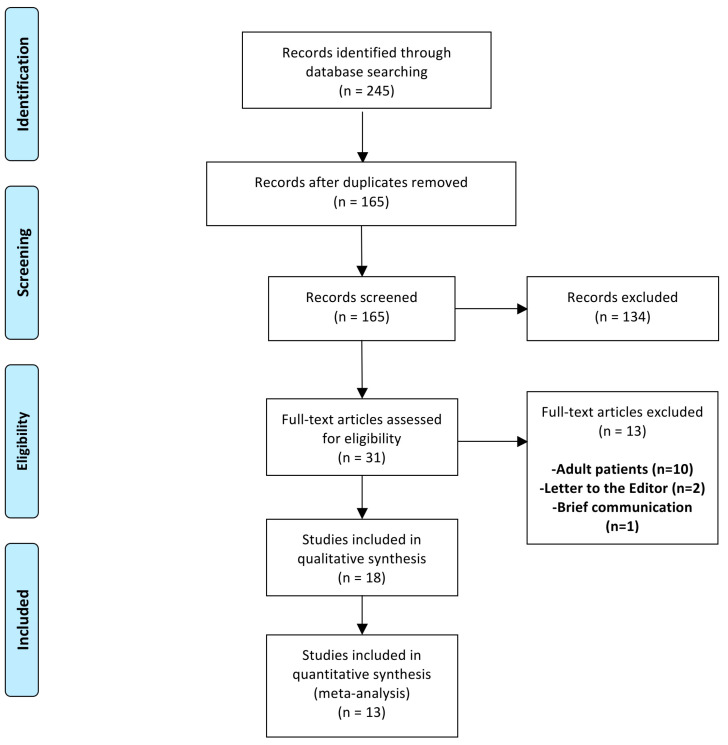
Selection of the relevant studies using the Preferred Reporting Items for Systematic Review and Meta-Analysis (PRISMA) flow diagram.

A total of 245 articles were identified with our search strategy. Of these, 80 duplicate records were removed. Subsequently, the abstracts of the remaining 165 articles were screened for eligibility (Figure 1). Of these, 134 abstracts were excluded and only 31 articles were eligible for full-text review. Thirteen of these were further excluded as they included only adult patients (*n* = 10), were letters to the editor (*n* = 2), or brief communication (*n* = 1). Although eighteen studies [30,31,32,33,34,35,36,37,38,39,40,41,42,43,44,45,46,47] were included for systematic review, only thirteen [30,31,32,33,34,35,36,37,38,39,40,41,42] were selected for the final meta-analysis. The remaining five studies [43,44,45,46,47] compared the two patient groups from non-identical time periods. A total of 2782 patients, 1239 and 1543 in groups A and B, respectively, were included in the meta-analysis.

The baseline characteristics of the studies included in the meta-analysis are demonstrated in Table 1. All studies, except one, were retrospective in nature. The study by Place et al. was cross-sectional in design [33]. Ten out of thirteen studies compared the average duration of symptoms among the two patient groups. A significant difference was observed in the four studies only [31,35,37,41]. Similarly, ten studies compared the average LOS among both groups [35,37,42].

### 3.2. Methodological Quality Assessment

The Downs and Black scoring by both authors is depicted in Table 2. The average scores ranged between 17.5 to 25. The total scores and inter-observer agreement are preselected in Table 3. Twelve out of thirteen studies had a moderate risk of bias. The studies by Place et al. [33] and Theodorou et al. [42] had the lowest and the highest scores, respectively. The inter-observer agreement was almost perfect (Kappa = 0.961; *p* < 0.001)

### 3.3. Outcome Analysis

#### 3.3.1. Proportion of Children with Complicated Appendicitis

**Figure 2:**
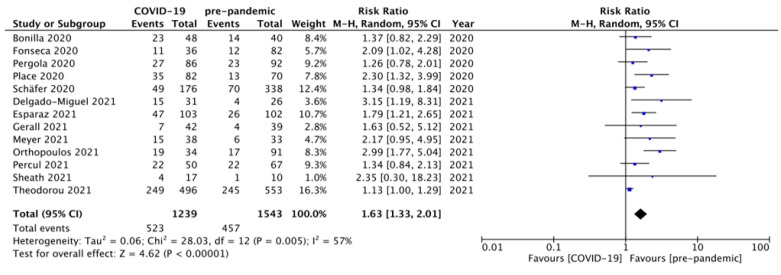
Forest plot comparison of the incidence of complicated appendicitis between the two groups of patients, i.e., presenting during the COVID-19 pandemic versus the pre-pandemic period. COVID—Coronavirus Disease 2019. M-H—Mantel–Haenszel method; CI—confidence interval.

This outcome was reported by all thirteen studies [30,31,32,33,34,35,36,37,38,39,40,41,42]. A total of 980 out of 2782 children, 523 and 457 belonging to groups A and B, respectively, developed complicated appendicitis. Pooling the data (Figure 2) demonstrated a statistically significant difference in the incidence of complicated appendicitis among the children of group A versus group B (RR = 1.63, 95% CI 1.33–2.01, *p* < 0.00001). For this outcome, the estimated heterogeneity among the included studies was statistically significant (I^2^ = 57%, *p* = 0.005).

#### 3.3.2. Proportion of Children Managed via the NOM

**Figure 3:**
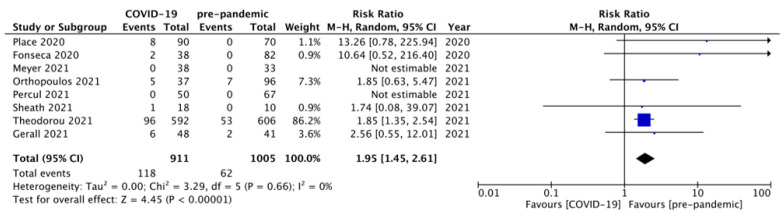
Forest plot comparison of the non-operative management (NOM) rates between the two groups of patients, i.e., presenting during the COVID-19 pandemic versus the pre-pandemic period. COVID—Coronavirus Disease 2019; M-H—Mantel–Haenszel method; CI—confidence interval.

Eight studies [31,33,37,38,39,40,41,42] compared the NOM rates among the children belonging to groups A versus B. Two of these had no patients managed via the NOM in either treatment group. Therefore, this outcome analysis consisted of events from six studies only. A total of 118/911 and 62/1005 children, belonging to groups A and B, respectively, were managed via the NOM. Pooling the data (Figure 3) demonstrated a significantly higher proportion of children managed via the NOM during the pandemic versus pre-pandemic period (RR = 1.95, 95% CI 1.45–2.61, *p* <0.00001). The estimated heterogeneity among the included studies was neither substantial nor statistically significant (*p* = 0.66; I^2^ = 0%) for this outcome.

## 4. Discussion

The aim of this study was to systematically summarize and measure the effects of the ongoing COVID-19 pandemic on the proportion of children with complicated appendicitis and those managed by NOM.

A classification of complicated appendicitis is given when there is evidence of a perforated or gangrenous appendix, an intra-abdominal abscess, or fecal peritonitis, which often results in a longer length of stay and greater rates of morbidity and mortality. Overall, complicated appendicitis is more common in children, with rates as high as 30% [6,48]. One of the reasons for the higher incidence of complicated appendicitis in young patients is diagnostic delay. The diagnostic delay is partly due to unclear anamnesis and atypical clinical presentations found in young patients. Studies showed that appendicitis is a diagnostic challenge with 7–15% of cases presenting twice to the emergency department before diagnosis, resulting in an increase in the rate of complications [49,50,51]. The risk of perforation within 24 h of the onset of symptoms is substantial (7.7%), and it increases in a linear fashion with duration, especially with prehospital delay, moreso than with admitted children [52]. Socioeconomic factors, which are globally worsened by ongoing COVID-19 pandemic, are also important factor in delayed presentation of pediatric patients as seeking medical care is dependent upon parents’ knowledge of illness, transportation options, insurance status, and financial wellbeing [53,54]. Another reason for higher incidence of complicated appendicitis in young patients is misdiagnosis. Misdiagnosis is due to the fact that the classical clinical symptoms and laboratory findings that are common in older pediatric population are missing in the younger [4]. Patient age is tied closely to the stage of acute appendicitis, so the youngest patients present with more advanced stages of disease and are at greater risk of perforation, with recent study showing a significant increase of perforation in relation with age as follows: 100%  <  1 year; 100% 1–2 years; 83.3% 2–3 years; 71.4% 3–4 years; 78.6% 4–5 years and 47.3% of  5 years [6]. Studies also demonstrate that using various clinical methods (clinical exam, laboratory tests, imaging and clinical scores), the availability of which can be reduced during COVID-19 pandemic, is associated with a reduction in the negative appendectomy rate from 14% to 4%, with a slight reduction in the rate of perforated appendicitis [55].

The most accepted mode of treatment of acute appendicitis is appendectomy following fluid resuscitation, analgesia, and intravenous antibiotics. Laparoscopic appendectomy is the most common surgical option with known benefits of lesser incidence of postoperative ileus, a shorter hospital stays, reduced analgesic requirements, a reduced incidence of wound infection and less risk of subsequent adhesive bowel obstruction [1,56,57,58,59]. Intra-abdominal abscess rates are similar after laparoscopic and open appendectomy and are largely determined by whether the appendix is perforated or not [1,56,57,58,59]. Another option for treatment is NOM (conservative therapy) which can represent a feasible option for acute appendicitis, although complication-free treatment success rates are higher with surgical treatment [1]. Systematic reviews suggest that NOM with antibiotics may fail during the primary hospitalization in about 8% of cases, and an additional 20% of patients might need a second hospitalization for recurrent appendicitis [60].

All of the aforementioned factors in diagnosis and treatment of pediatric appendicitis are being affected by the ongoing COVID-19 pandemic. Since it started in March of 2020, the COVID-19 pandemic represents significant global health threat, a political challenge and has severely affected human life and welfare [11,61]. Extensive measures, most significant being lockdowns, have been implemented to lower person-to-person transmission and to stop distribution of virus. In the beginning of the pandemic, lockdowns and “Staying home” were most common means to prevent transmission of the virus. During the COVID-19 pandemic elective surgical procedures were canceled in most centers. Surgical procedures were limited only for the care of urgent surgical patients [12,13,14,15,16,17,62,63]. These efforts to minimize unnecessary traffic through the healthcare facility resulted in a significant reduction in emergency department patient encounters [61,63].

Results from our study demonstrate a significantly higher incidence of complicated appendicitis among the children in pandemic group versus non-pandemic group. The same results were reported by nine studies included in the meta-analysis, while another four [32,40,41,42,43] showed an increase in complicated appendicitis but with no statistical significance. Results from six studies, included in secondary outcome, demonstrate a significantly higher proportion of children managed via the NOM during the pandemic versus pre-pandemic period [31,33,37,39,41,42]. A recent study by Lazzerini et al. found that pediatric emergency department visits in Italy were reduced by as much as 88% during the peak of their COVID-19 outbreak, in comparison with pre-pandemic years [64].

Significantly higher complicated appendicitis rates during the pandemic can be explained by multiple factors. Delayed presentation of pediatric patients, in general, and higher incidence of NOM during pandemic, are the most important ones. Socioeconomics and delay in time from admission to surgery because of pandemic protocols could be speculated as minor factors [30]. As previously mentioned, the risk of perforation and other complications increases in a linear fashion with duration of disease, especially with pre-hospital delay more than with admitted children. Studies included in the meta-analysis had different findings in regard to delays from the onset of symptoms to admission at the emergency department. Several studies recorded longer prehospital delay in admission of acute appendicitis during the pandemic [9,31,35,37,38,41], while other studies showed no significant difference between pandemic and pre-pandemic delay of presentation [31,34,36,38,40,42]. Significant increases in delayed care for different medical emergencies, including pediatric surgical emergencies, during the COVID-19 pandemic period have been noted by the medical community and published in several reports [65]. The effects of the COVID-19 pandemic are recorded in other urgent pediatric surgery conditions such as testicular torsion, in which latest studies show significantly longer time from testicular torsion symptom onset to presentation during the pandemic and a significantly higher proportion of patients reported delaying care [17,66]. Recent studies show that the outbreak of the COVID-19 pandemic is associated with a delay in presentation of patients with most common medical emergencies such as acute ischemic stroke and delay of diagnosis of colorectal carcinomas, which will lead to a massive downstream impact on healthcare [67,68]. Delayed presentation can be explained by avoidance of unnecessary hospital visits in the absence of severe symptoms and reduced or delayed access to medical care due to parental fear of children’s exposure to COVID-19.

As per the findings from recent adult/pediatric studies, the patients developing appendicitis during the pandemic reach healthcare facilities on time (similar to the pre-pandemic period). Although an identical management algorithm of acute appendicitis was followed during the two time periods, more reliance on non-operative management was observed among the surgeons during the COVID-19 pandemic [69,70]. Our analysis shows the same result. The main reasons for NOM were the risk of false negative testing and prevention of viral transmission to healthcare workers in the operating room as well as to minimize hospital resource utilization. Additionally, it could be speculated that more patients asked for non-surgical treatment strategies during the pandemic as compared with the cases before the outbreak, with the fear of hospital admission and acquiring the COVID-19 infection from the hospital.

Open surgery is suggested as a possible approach because of the shorter operation time and lower risk of COVID-19 transmission [71,72]. Widespread use of laparoscopic approach and surgeons not being familiar with open surgery could be a reason for higher incidence and more reliance on NOM during pandemic.

Fonseca et al. reported a 56% reduction in the number of appendectomies performed in pandemic group in comparison with pre-pandemic, and Percul et al. reported a reduction of 25% in total number of acute appendicitis cases [31,40]. It could be speculated that during COVID-19 pandemic, patients with mild or non-specific symptoms were not seeking medical care because of the concern about acquiring COVID-19 infection. The number of cases that resolved on their own or are treated with antibiotics prescribed by gatekeepers should also be considered. Confounding variables such as movement restrictions, difference between mild and strict lockdown restrictions, travel restrictions, limited resources, studies being researched mainly in tertiary centers, and other pandemic-induced changes should also be acknowledged [36]. Here additional research is needed and data from outpatient medical care needs to be researched.

The results of this study clearly demonstrated a significantly higher number of complicated appendicitis during the COVID-19 pandemic compared with pre-COVID-19 period. Additionally, the study demonstrates an increase in NOM of appendicitis during the pandemic. Both outcomes are direct effect of the COVID-19 pandemic. Management of pediatric appendicitis during this pandemic must be evaluated individually for every hospital and its capacity for SARS-CoV-2 testing, laboratory tests, imaging options, bed, staff, emergency ward capacity and personal protective equipment capacity. Although appendectomy should not be impacted by restrictions on elective procedures several institutions, countries and professional associations recommend performing NOM for appendicitis during pandemic [73].

COVID-19 fundamentally changes the way emergency wards and hospitals function and deliver patient care. The overflow of COVID-19 patients and the effects of the pandemic on health systems, in general, influenced emergency and pediatric specialties wards, which condensed as both elective and emergency care across pediatric specialties decreased and many of these wards were converted to adult wards to accommodate the overflow of adult COVID-19 patients. There were numerous considerations and limitations to consider while delivering patient care, attempting to limit hospital stays while also limiting the number of operations. In conclusion, COVID-19 is a global pandemic, challenging healthcare systems worldwide. During these challenging times, we address the importance of a comprehensive evaluation, physical examination, appropriate and effective treatment in children suspected of having any surgical condition. Balance should be achieved between measures designed to end the pandemic and the appropriate care of pediatric population requiring surgical care.

The results of this review must be interpreted within the context of few limitations. First, all except one of the included comparative studies had a moderate risk of bias. Second, the retrospective nature of all studies, except one, is a source of information bias due to variable reporting. Only eight studies reported the proportion of children managed via the NOM. A similar variable reporting was observed in terms of the baseline characteristics of the included studies. The average duration of symptoms and the average hospital stay were non-uniformly reported (both in terms of the averages and the means of dispersion). Third, this meta-analysis involves the pooling of heterogeneous data (I^2^ >50% for the primary outcome). Although we excluded the studies where patients were managed during non-identical months of the pandemic and pre-pandemic periods, the study durations were still not constant across all the included studies. Some other factors that might have contributed to the heterogeneity are the inclusion of the subjects from different geographical locations, variations in socioeconomics in different parts of the world, etc. Finally, these patients were managed by different surgeons in different healthcare facilities. Differences in the management protocols at these centers might have affected the appendectomy and NOM rates.

## 5. Conclusions

The results of this meta-analysis depict a significantly higher incidence of complicated appendicitis in children during the COVID-19 pandemic than in the pre-COVID-19 period. Additionally, a significantly higher proportion of children were managed via the NOM during the pandemic in comparison to the pre-pandemic period. However, the moderate risk of bias of majority of the included studies prevents us from deriving an appropriate estimate of the overall effect. Further studies with more homogeneous study samples need to be conducted before a definite conclusion is drawn.

## Figures and Tables

**Table 1:** Baseline characteristics of the studies included in meta-analysis.

Author	Study Design	Sample Size		Average (Mean/Median) Duration of Symptoms; Hours		Average (Mean/Median) LOS; Days	
		A	B	A	B	A	B
Bonilla et al., 2020 [30]	Retro	49	41	40 (11–301) *	30.5 (9–248) *	5.3 (6.7) ^†^	4.2 (4.3) ^†^
Fonseca et al., 2020 [31]	Retro	38	82	40.6 (35.5) ^†^	28.2 (23.2) ^†^	2.2 (2.0) ^†^	2.4 (2.6) ^†^
La Pergola et al., 2020 [32]	Retro	86	92	1 (1–2) * days	1 (1–2) * days	Not mentioned	
Place et al., 2020 [33]	Cross	90	70	Not mentioned		Not mentioned	
Schäfer et al., 2020 [34]	Retro	176	338	Not mentioned		5.3 (0.2) ^†^	4.9 (0.1) ^†^
Delgado-Miguel et al., 2021 [35]	Retro	31	26	46.8 (13.5) ^†^	22.9 (11.5) ^†^	4.9 (3.2) ^†^	2.5 (1.4) ^†^
Esparaz et al., 2021 [36]	Retro	103	102	Not mentioned		Not mentioned	
Gerall et al., 2021 [37]	Retro	48	41	2 (0.5–14) * days	1 (0.5–14) * days	2 (0.5–22) *	1 (0.5–9) *
Meyer et al., 2021 [38]	Retro	38	33	2 days *	2 days *	6 days *	6 days *
Orthopoulos et al., 2021 [39]	Retro	37	96	Not mentioned		2.5 (3.1) ^†^	1.8 (1.9) ^†^
Percul et al., 2021 [40]	Retro	50	67	24 (3–120) *	24 (4–96) *	3 (2) ^†^ days	3 (2) ^†^ days
Sheath et al., 2021 [41]	Retro	18	10	4 (1–14) * days	2 (1–10) * days	1 (0–10) *	1 (0–6) *
Theodorou et al., 2021 [42]	Retro	592	606	2 (1–3) * days	2 (1–3) * days	3 (1–5) *	2 (1–4) *

^†^ Mean (SD); * Median (range); Abbreviations: Retro—retrospective study; Cross—cross-sectional stud; A—group A (pandemic group); B—group B (pre-pandemic group); LOS—length of stay; Significant difference (*p* < 0.05) represented by shaded cells.

**Table 2:** Downs and Black scale scores for the included studies by Observer 1 and Observer 2.

Study	Reporting	External Validity	Internal Validity-Bias	Internal Validity-Confounding	Power	Total Scores
Methodological assessment by Observer 1						
Orthopoulos, 2021 [39]	9	3	5	2	0	19
Bonilla, 2021 [30]	8	3	5	2	0	18
Gerall, 2021 [37]	10	3	5	2	0	20
Fonseca, 2020 [31]	10	3	5	2	0	20
Sheath, 2021 [41]	9	3	5	2	0	19
Place, 2020 [33]	8	3	5	2	0	18
Percul, 2021 [40]	10	3	5	2	0	20
Delgado-Miguel, 2021 [35]	9	3	5	2	0	19
Meyer, 2021 [38]	8	3	5	2	0	18
Esparaz, 2021 [36]	8	3	5	2	0	18
Pergola, 2020 [32]	8	3	5	2	0	18
Schafer, 2021 [34]	8	3	5	2	0	18
Theodorou, 2021 [42]	11	3	5	2	4	25
Methodological assessment by Observer 2						
Orthopoulos, 2021 [39]	10	3	5	2	0	20
Bonilla, 2021 [30]	9	3	5	2	0	19
Gerall, 2021 [37]	10	3	5	2	0	20
Fonseca, 2020 [31]	9	3	5	2	0	19
Sheath, 2021 [41]	9	3	5	2	0	19
Place, 2020 [33]	7	3	5	2	0	17
Percul, 2021 [40]	10	3	5	2	0	20
Delgado-Miguel, 2021 [35]	10	3	5	2	0	20
Meyer, 2021 [38]	8	3	5	2	0	18
Esparaz, 2021 [36]	8	3	5	2	0	18
Pergola, 2020 [32]	9	3	5	2	0	19
Schafer, 2021 [34]	8	3	5	2	0	18
Theodorou, 2021 [42]	11	3	5	2	4	25

**Table 3:** The total scores and inter-observer agreement.

Study	Rater 1	Rater 2	Mean	Kappa Value	*p*
Orthopoulos, 2021 [39]	19	20	19.5	0.961	<0.001
Bonilla, 2021 [30]	18	19	18.5		
Gerall, 2021 [37]	20	20	20		
Fonseca, 2020 [31]	20	19	19.5		
Sheath, 2021 [41]	19	19	19		
Place, 2020 [33]	18	17	17.5		
Percul, 2021 [40]	20	20	20		
Delgado-Miguel, 2021 [35]	19	20	19.5		
Meyer, 2021 [38]	18	18	18		
Esparaz, 2021 [36]	18	18	18		
Pergola, 2020 [32]	18	19	18.5		
Schafer, 2021 [34]	18	18	18		
Theodorou, 2021 [42]	25	25	25		

## Data Availability

The data presented in this study is available upon request of the respective author.

## Appendix A

PUBMED—((“appendical” [All Fields] OR “appendicitis” [MeSH Terms] OR “appendicitis” [All Fields]) AND (“covid 19” [MeSH Terms] OR “covid 19” [All Fields] OR “covid19” [All Fields]) AND “LitCTREATMENT” [Filter] AND (“infant” [MeSH Terms] OR “child” [MeSH Terms] OR “adolescent” [MeSH Terms])) AND (allchild [Filter]).

EMBASE—appendicitis:ti,ab,kw AND (coronavirus:ti,ab,kw OR ‘covid 19’:ti,ab,kw) AND ([adolescent]/lim OR [child]/lim).

Web of science—Query 1: ((ALL = (coronavirus)) OR ALL = (SARS-CoV-2)) OR ALL =(COVID-19)) OR ALL = (novel coronavirus) AND Query 2: ALL = (appendicitis). Refine search using keyword “children”.

SCOPUS—(TITLE-ABS-KEY (appendicitis) AND TITLE-ABS-KEY (covid19 OR coronavirus OR SARS-CoV-2) AND TITLE-ABS-KEY (children OR pediatric).

**Table A1:** Detailed search strategy.

Database	Studies
PubMed	66
EMBASE	102
Web of science	54
SCOPUS	123
Total	345
Duplications	180
After duplication removal	165
